# Safety and efficacy of tolvaptan in real‑world Japanese patients with autosomal dominant polycystic kidney disease: final results of SLOW‑PKD surveillance

**DOI:** 10.1007/s10157-025-02634-7

**Published:** 2025-02-14

**Authors:** Toshio Mochizuki, Satoru Muto, Kyoko Suzue, Satoshi Komaniwa, Toshiki Tanaka, Yasuhiko Fukuta, Yuko Yamashige

**Affiliations:** 1 Department of Nephrology, Tokyo Women’s Medical University, 8-1 Kawada-Cho, Shinjuku-Ku, Tokyo, 162-8666 Japan; 2 Clinical Research Division for Polycystic Kidney Disease, Department of Nephrology, Tokyo Women’s Medical University, 8-1 Kawada-Cho, Shinjuku-Ku, Tokyo, 162-8666 Japan; 3 Present Address: PKD Nephrology Clinic, Tokyo, Japan; 4 Department of Urology, Graduate School of Medicine, Juntendo University, Tokyo, Japan; 5 Department of Advanced Informatics for Genetic Disease, Graduate School of Medicine, Juntendo University, Tokyo, Japan; 6 Present Address: Department of Urology, Juntendo University Nerima Hospital, Tokyo, Japan; 7 Department of Pharmacovigilance, Otsuka Pharmaceutical Co., Ltd, 3-2-27 Otedori, Chuo-Ku, Osaka, 540-0021 Japan; 8 Department of Medical Affairs, Otsuka Pharmaceutical Co., Ltd, 2-16-4 Konan, Minato-Ku, Tokyo, 108-8241 Japan

**Keywords:** Autosomal dominant polycystic kidney disease, Post-marketing survey, Glomerular filtration rate, Total kidney volume, Safety profile, Tolvaptan

## Abstract

**Background:**

Tolvaptan, a vasopressin type 2 receptor antagonist, has been used to treat autosomal dominant polycystic kidney disease in Japan since 2014.

**Methods:**

This long-term, real-world, post-marketing surveillance (PMS) was conducted in Japan from March 2014 to March 2022. Safety was assessed based on adverse drug reactions (ADRs). For efficacy, changes in the slope of total kidney volume (TKV) and estimated glomerular filtration rate (eGFR) were assessed before and during the administration of tolvaptan.

**Results:**

A total of 1676 patients were enrolled, with mean TKV (n = 1000) of 2149 ± 1339 mL and eGFR (n = 1641) of 44.4 ± 21.7 mL/min/1.73 m^2^. Frequent ADRs were hepatic function abnormal (9.6%), hyperuricaemia (8.3%), and thirst (8.1%). Most of the increased alanine aminotransferase exceeding 3 times the upper limit of the reference level occurred from 3  to  14 months after the start of treatment, but about 20% was observed after 15 months. There was no increase in ADRs over 36 months, suggesting that no other safety concerns need to be monitored during administration over 3–7 years. The mean slope of the estimated TKV increase before and during tolvaptan treatment was 6.58 and 3.71%/year, respectively (*P* = 0.0020). The mean slope of eGFR decline was − 3.63 and − 3.26 mL/min/1.73 m^2^/year, respectively (*P* = 0.2728).

**Conclusion:**

There were no major problems with the safety of tolvaptan treatment, and efficacy in limiting TKV increase in this PMS was comparable to the previous, pivotal randomized control trials.

*Trial registration* ClinicalTrials.gov; NCT02847624.

**Supplementary Information:**

The online version contains supplementary material available at 10.1007/s10157-025-02634-7.

## Introduction

Autosomal dominant polycystic kidney disease (ADPKD) is the most common hereditary monogenic kidney disorder and the fourth leading cause of end-stage kidney disease (ESKD) in adults worldwide [1, 2]. ADPKD occurs in all races worldwide [2] and its prevalence is estimated to be 1/4000 in Japan [3], and less than 5/10,000 in Europe [4]. The clinical hallmark of ADPKD is the development of fluid-filled renal cysts leading to organ enlargement, chronic kidney disease (CKD), and extra-renal complications such as hypertension, liver cysts and intracranial aneurysms [5–7].

Tolvaptan, an oral selective vasopressin V2 receptor antagonist, decreases fluid secretion and cell proliferation, thus slowing ADPKD progression [8, 9]. A phase 3 clinical trial, Tolvaptan Efficacy and Safety in Management of Autosomal Dominant Polycystic Kidney Disease and Its Outcomes (TEMPO 3:4), was conducted to evaluate the efficacy and safety of tolvaptan over 3 years [10]. The study included 1445 patients with ADPKD with a total kidney volume (TKV) of ≥ 750 mL and a creatinine clearance (CCr) ≥ 60 mL/min. The results showed treatment with tolvaptan reduced the annual increase in TKV and slowed annual estimated glomerular filtration rate (eGFR) decline, compared to placebo [10]. Subsequently, in 2014, Japan became the first country in the world to approve tolvaptan for the treatment of patients with ADPKD. Furthermore, the REPRISE study (Replicating Evidence of Preserved Renal Function: An Investigation of Tolvaptan Safety and Efficacy) confirmed that tolvaptan was effective in patients with later stage ADPKD [11]. In Japan’s practical guidelines for PKD, tolvaptan has been recommended as a grade 1A treatment in patients whose condition is expected to progress rapidly [12]. Since 2014, post-marketing surveillance (PMS), termed SLOW-PKD surveillance (Samsca^®^ Long-term surveillance of tolvaptan in PKD patients in real-world setting), has been conducted to evaluate the safety and efficacy of tolvaptan in real-world clinical settings in Japan and the first five-year interim results have been reported [13]. Here we report the final results of the SLOW-PKD surveillance.

## Materials and methods

### Surveillance design

This was a prospective, multicenter, observational, eight-year PMS to evaluate the long-term safety and efficacy of tolvaptan in Japan from March 2014 to March 2022.

This surveillance has been performed in compliance with Good Post-marketing Study Practice (GPSP). Informed consent and ethics committee approval were not required under the GPSP and were accordingly not mandatory during this surveillance.

### Surveillance population

Patients who met the diagnostic criteria for ADPKD [14] were registered in this survey. TKV of more than 750 mL and an annual TKV-slope increase of more than 5% as measured by magnetic resonance imaging (MRI), computed tomographic (CT) scanning, or ultrasound were included. Patients with contra-indications for the use of tolvaptan, based on the package insert, included those with serious renal impairment (eGFR of less than 15 mL/min/1.73 m^2^), liver injury, hypernatraemia, difficulties with water intake, or pregnancy, and were excluded.

### Data collection

The main data collected were demographic characteristics before tolvaptan treatment; TKV; kidney function (serum creatinine, eGFR); other laboratory values, especially those related to liver function; adverse events (AEs) and CKD stage [15].

### Safety assessment

All events identified as AEs were aggregated, regardless of their causal relationship with tolvaptan therapy. The events for which a causal relationship to tolvaptan could not be ruled out by the attending physicians were categorized as adverse drug reactions (ADRs). AEs defined as “of special interest” included acute hepatic failure, impaired liver function, thirst, hypernatraemia, blood sodium increased, central pontine myelinolysis, dehydration, thrombosis, thromboembolism, renal failure, renal disorder, gout, hyperuricaemia, dizziness, diabetes, hyperglycaemia, glaucoma, syncope, loss of consciousness, hyperkalaemia, blood potassium increased, shock, anaphylaxis, excessive blood pressure reduction, ventricular fibrillation, and ventricular tachycardia. The priority items to be investigated were acute liver failure, hepatic dysfunction, and hypernatraemia. Significant potential risks were drug interactions (in combination with cytochrome P450 [CYP3A4] inhibitors) and skin neoplasm. It was mandatory for liver function to be monitored at least once every month. Liver function was assessed based on alanine aminotransferase (ALT) levels. Abnormal levels were categorized based on the maximum ALT levels: > 30 and ≤ 90 IU/L, > 90 and ≤ 240 IU/L, and > 240 IU/L. The ALT upper limit of normal (ULN) was set at more than 30 IU/L in accordance with the insurance guidance program issued by Japan’s Ministry of Health, Labour and Welfare (MHLW) in 2018.

### Efficacy assessment

TKV was measured by each attending physician based on MRI or CT imaging. Slope was analyzed in patients who had at least one pre-baseline (pre-treatment), one baseline, and one post-baseline (post-treatment) measurement (one month or more after starting tolvaptan treatment). The eGFR was measured at each site, and these data were used for estimating slopes in patients who had at least one pre-baseline and one post-baseline measurement. Data collected within one month after the start of treatment were excluded from the analysis since the hemodynamic effect was reported after three weeks in patients with ADPKD [16]. The impact of the initial eGFR dip was not excluded in the efficacy assessment, since this was an observational survey that collected real-world data.

### Statistical analysis

The target number of patients to be enrolled was set at 1600 in order to detect ADRs with an incidence of 0.3% or more, providing statistical power of ≥ 99%. In addition, assuming that the continuous administration rate for more than 4 years was 50% (approximately 800 of patients), therefore, we considered that it was possible to collect data on 800 patients treated with tolvaptan for over 4 years.

Summary statistics were calculated and the number of patients, composition ratio, and incidence rate were calculated by frequency aggregation. Testing was at a two-sided significance level of 5%, and two-sided 95% confidence intervals (CIs) were used.

AEs were coded using Medical Dictionary for Regulatory Activities (MedDRA) version 25.1. A multivariate logistic regression analysis was performed using factors that may affect clinical practice as explanatory variables, “the presence or absence of ADRs related to liver dysfunction” used as the dependent variable.

In efficacy analyses of TKV and eGFR, we used a mixed model with fixed effects of treatment, time and subject, interaction of treatment and time, interaction of subject and time as covariates, and random effects of intercept and time for patients with both pre-baseline (pre-treatment) and treatment values. In the model, treatment was an indicator of pre-treatment or treatment observations, and time was the period in days calculated from pre-baseline to baseline observations for the pre-treatment period, and from baseline to post-baseline observations for the treatment period. An unstructured variance–covariance matrix was assumed for the mixed model. Observations of the eGFR were analyzed directly in the mixed model, however, log_10_ transformations of TKV were analyzed using the mixed model, and anti-log_10_, with subtraction from 1 followed by multiplication by 100, was applied to convert the estimates and their 95% CIs to a scale of percentage annual change in baseline TKV. The mean estimated slope were calculated for each pre-treatment and treatment period in terms of the rate of change in TKV and eGFR. The difference in the estimated slope between the pre-treatment and treatment periods and use of the Wald test showed the estimated value of the parameter based on the solution of the fixed effect between the treatment group and the time, with a *P*-value based on the t-distribution.

## Results

### Patient disposition and baseline characteristics

**Fig. 1:**
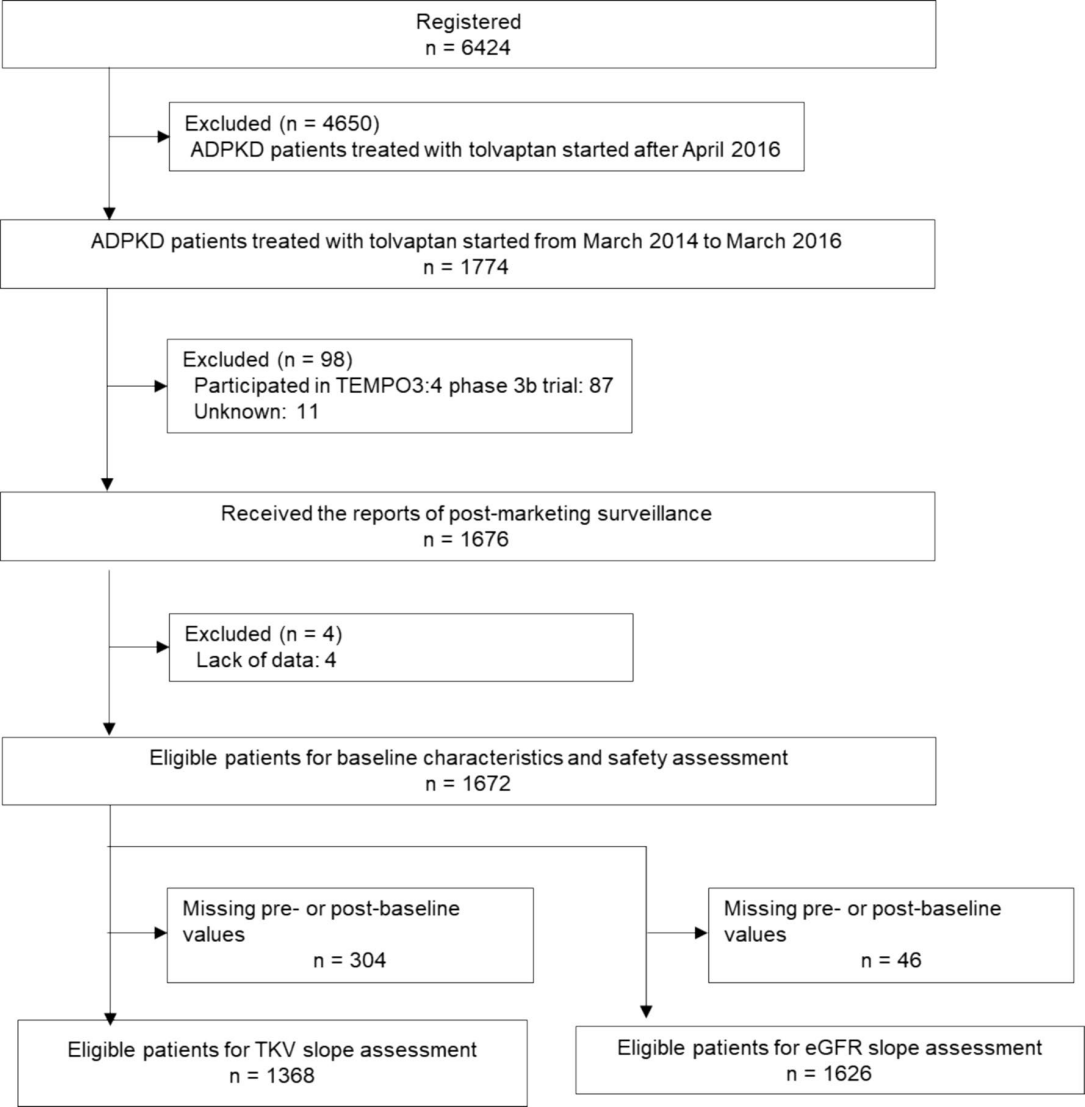
Flow chart of the surveillance population. ADPKD, Autosomal dominant polycystic kidney disease; eGFR, estimated glomerular filtration rate; TKV, total kidney volume

This PMS was conducted at 902 sites in Japan between March 2014 and March 2022. Among the 6424 patients registered, a total of 1774 patients, whose treatment with tolvaptan was started between March 24 2014 and March 31 2016 and in whom case report forms were recovered, were enrolled (Fig. 1). The median follow-up period was 1461 (range, 1—2885) days. From the safety and efficacy assessments, the baseline characteristics of 1672 patients treated with tolvaptan are shown in Table 1. The mean ± standard deviation (SD) of baseline eGFR (n = 1641) and TKV (n = 1000) was 44.4 ± 21.7 mL/min/1.73 m^2^ and 2149 ± 1339 mL, respectively. Approximately half of the patients had CKD stage G3b (n = 452, 27.0%) and G4 (n = 501, 30.0%). Baseline characteristics of patients eligible for TKV analysis at each CKD stage (n = 1670) are shown in Supplementary Table S1.

**Table 1:** Patients characteristics

	N = 1672
Gender	
Male, n (%)	867 (51.9)
Female, n (%)	805 (48.1)
Age (years)	
Mean ± SD (n)	49.7 ± 11.2 (1672)
Weight (kg)	
Mean ± SD (n)	64.2 ± 12.7 (1404)
Body mass index (kg/m^2^)	
Mean ± SD (n)	23.3 ± 3.5 (1373)
Blood urea nitrogen (mg/dL)	
Mean ± SD (n)	23.9 ± 9.5 (1628)
Serum creatinine (mg/dL)	
Mean ± SD (n)	1.5 ± 0.7 (1641)
eGFR (mL/min/1.73 m^2^)	
Mean ± SD (n)	44.4 ± 21.7 (1641)
G1, n (%)	63 (3.8)
G2, n (%)	308 (18.4)
G3a, n (%)	336 (20.1)
G3b, n (%)	452 (27.0)
G4, n (%)	501 (30.0)
G5, n (%)	10 (0.6)
Total kidney volume (mL)	
Mean ± SD (n)	2149 ± 1339 (1000)
< 750 mL, n (%)	5 (0.3)
≥ 750– < 1500 mL, n (%)	385 (23.0)
≥ 1500– < 3000 mL, n (%)	417 (24.9)
≥ 3000– < 4500 mL, n (%)	142 (8.5)
≥ 4500– < 6000 mL, n (%)	31 (1.9)
≥ 6000 mL, n (%)	20 (1.2)
Missing or before Day −90, n (%)	672 (40.2)
Height-adjusted total kidney volume (mL/m)	
Mean ± SD (n)	1241 ± 762 (1389)
Complications	
Hypertension, n (%)	1447 (91.0)
Diabetes, n (%)	74 (4.7)
Hyperlipidaemia, n (%)	460 (28.9)
Hyperuricaemia, n (%)	746 (46.9)
Liver disease, n (%)	758 (47.7)
Cystic liver, n (%)	739 (46.5)
Others, n (%)	54 (3.4)
Pancreatic cysts, n (%)	19 (1.2)
Cerebral aneurysm, n (%)	161 (10.1)
Kidney disease, n (%)	254 (16.0)
Urinary calculus, n (%)	36 (2.3)
Cyst infection, n (%)	26 (1.6)
Urinary tract infection, n (%)	7 (0.4)
Others, n (%)	786 (49.4)
Physical findings (symptoms)	
Low back pain / flank pain including nephralgia, n (%)	249 (16.0)
Feeling of abdominal distension, n (%)	408 (26.4)
Anorexia, n (%)	74 (12.4)
General malaise, n (%)	90 (15.2)
Hematuria, n (%)	105 (6.8)

*eGFR* estimated glomerular filtration rate

### Treatment with tolvaptan

The starting dose of tolvaptan is summarized in Table 2. The mean ± SD of tolvaptan dose at the start of surveillance was 47.2 ± 17.8 mg/day (n = 1672) (data not shown). The mean treatment period was 1320 ± 765 days (n = 1667). During this period, 663 patients were discontinued from tolvaptan administration due to adverse events (n = 318), lack of efficacy (n = 60), no visit to the site (n = 28), and other reasons (unspecified) (n = 272) including multiple reasons.

**Table 2:** Starting dose by CKD stage

CKD stage, n (%)	Starting dose		
	< 60 mg	60 mg	> 60 mg
G1	12 (0.7)	51 (3.1)	0 (0.0)
G2	81 (4.8)	227 (13.6)	0 (0.0)
G3a	95 (5.7)	240 (14.4)	1 (0.1)
G3b	157 (9.4)	295 (17.6)	0 (0.0)
G4	293 (17.5)	208 (12.4)	0 (0.0)
G5^a^	6 (0.4)	4 (0.2)	0 (0.0)
Unknown	0 (0.0)	2 (0.1)	0 (0.0)
Total	644 (38.5)	1027 (61.4)	1 (0.1)

^a^Administration to patients with an eGFR less than 15 is contraindicated in the package insert. However, in this real-world study, patients with CKD stage G5 were enrolled and received tolvaptan

*CKD* Chronic kidney disease

### Safety

**Fig. 2:**
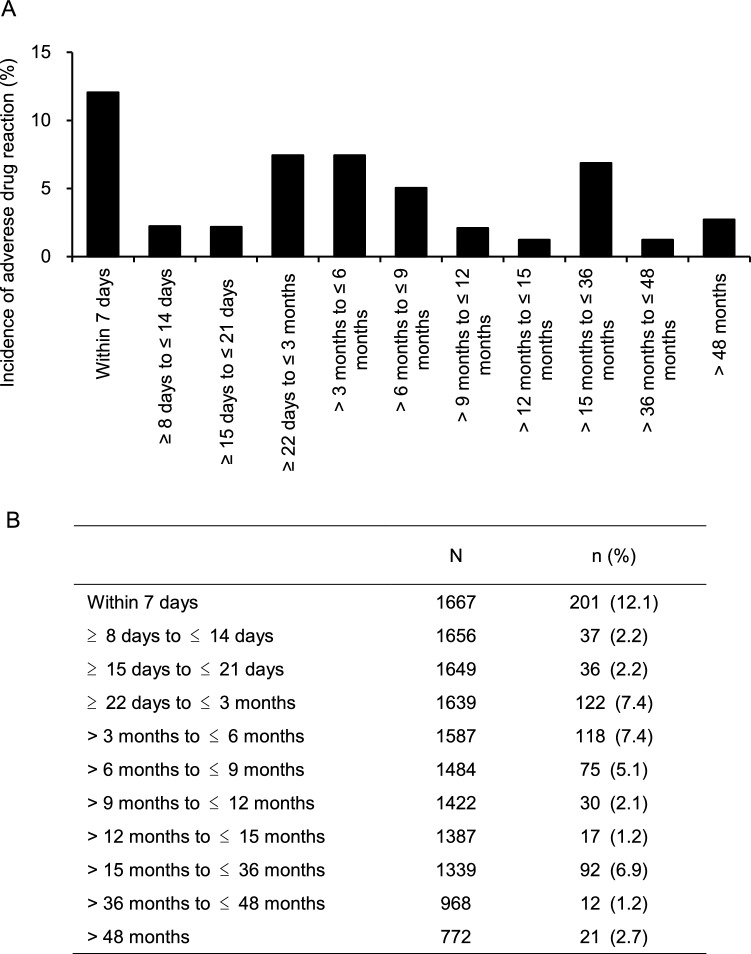
Onset time of adverse drug reactions. **A** incidence (%) of adverse drug reaction per time, **B** total number of patients, number (%) of patients who experienced drug adverse reaction per time

**Fig. 3:**
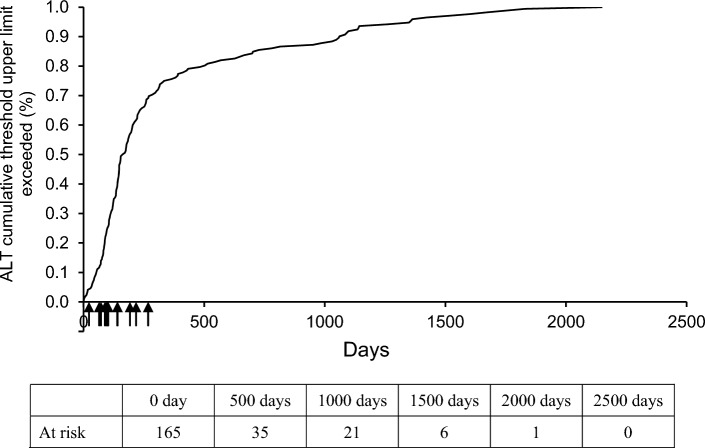
The period until the ALT first became three-fold greater than the upper limit of the reference value, Kaplan–Meier curve with 1.0 for all cases that reached or exceeded triple the reference value. The 14 cases where relationship between liver function-related impairment and tolvaptan administration was considered “definite”, “highly likely”, and “probable” by the Hepatic Adjudication Committee (post-administration onset date: on days 22, 64, 72, 88, 94, 98, 100, 102, 139, 141, 192, 217, 267, and 268) are shown in ↑. ALT, alanine transaminase

The incidence of any AE was 1086 (65.0%) in 1672 patients. The serious AE (SAEs) incidence was 253 (15.1%) in 1672 patients (data not shown). The incidence of ADRs was 777 (46.5%) in 1672 patients. ADRs with an incidence of ≥ 1.0% were shown in Table 3. Serious ADRs were reported in 83 (5.0%) of 1672 patients; hepatic function abnormal (0.8%), liver disorder (0.5%), renal cyst infection (0.3%), renal failure (0.3%), drug-induced liver injury (0.2%), renal cyst haemorrhage (0.2%), and renal impairment (0.2%) (Supplementary Table S2). Table 4 indicates the ADRs of special interest. The onset times of ADRs and ADRs of special interest are shown in Fig. 2 and Supplementary Figs. S1–S12. The incidence of ADRs between 36 and 48 months was 1.2% (12/968 patients), and after more than 48 months was 2.7% (21/772 patients). ALT increase to over 30 IU/L was observed in 571 of 1492 patients (38.3%) whose ALT levels were below 30 IU/L at baseline (Table 5). Of these, ALT increased between 30 and 90 IU/L was observed in 442 patients (29.6%), between 90 and 240 IU/L in 91 patients (6.1%), and over 240 IU/L in 38 patients (2.5%). Most of the increased ALT exceeding three times the upper limit of the reference level occurred during the 3 − 14 months after the start of treatment with tolvaptan, but about 20% was observed after 15 months (427 days) (Fig. 3). Incidence of liver-related ADRs by CKD stage is shown in Supplementary Fig. S13. Multivariate logistic regression analysis showed that the baseline CKD stage (adjusted odds ratio per unit: 0.772, 95% CI 0.643–0.926) and complications/liver disease (adjusted odds ratios for none: 1.683, 95% CI 1.135–2.495) were significant factors affecting hepatic disorders (Supplementary Table S3).

**Table 3:** Adverse drug reactions (> 1.0%)

	n (%)
Number of patients	1672
Any adverse drug reactions	777 (46.5)
Hepatic function abnormal	160 (9.6)
Hyperuricaemia	138 (8.3)
Thirst	135 (8.1)
Hypernatraemia	93 (5.6)
Liver disorder	53 (3.2)
Renal impairment	53 (3.2)
Pollakiuria	31 (1.9)
Gamma-glutamyltransferase increased	22 (1.3)
Blood creatinine increased	21 (1.3)
Dizziness	21 (1.3)
Nocturia	20 (1.2)
Dehydration	20 (1.2)
Constipation	19 (1.1)
Hyperkalaemia	18 (1.1)
Blood urea increased	17 (1.0)
Hepatic enzyme increased	16 (1.0)
Nausea	16 (1.0)
Insomnia	16 (1.0)

MedDRA Ver. 25.1

**Table 4:** Adverse drug reactions of special interest

Safety specification preferred terms	n (%)
Number of patients	1672
Acute hepatic failure and Hepatic function disorder^a^	262 (15.7)
Acute hepatic failure	1 (0.1)
Alanine aminotransferase abnormal	1 (0.1)
Alanine aminotransferase increased	9 (0.5)
Aspartate aminotransferase abnormal	1 (0.1)
Aspartate aminotransferase increased	13 (0.8)
Gamma-glutamyltransferase increased	22 (1.3)
Hepatic function abnormal	160 (9.6)
Hepatic steatosis	1 (0.1)
Hepatitis acute	1 (0.1)
Hyperbilirubinaemia	2 (0.1)
Liver disorder	53 (3.2)
Liver function test abnormal	2 (0.1)
Transaminases increased	1 (0.1)
Hepatic enzyme increased	16 (1.0)
Drug-induced liver injury	11 (0.7)
Hepatic cancer	1 (0.1)
Liver function test increased	3 (0.2)
Thirst	135 (8.1)
Lip dry	1 (0.1)
Thirst	135 (8.1)
Hypernatraemia	102 (6.1)
Blood sodium increased	10 (0.6)
Hypernatraemia	93 (5.6)
Renal Failure and Impairment	105 (6.3)
Blood creatinine abnormal	1 (0.1)
Blood creatinine increased	21 (1.3)
Blood urea increased	17 (1.0)
Glomerular filtration rate decreased	3 (0.2)
Proteinuria	3 (0.2)
Renal disorder	2 (0.1)
Renal failure	11 (0.7)
Protein urine present	2 (0.1)
Postrenal failure	1 (0.1)
Renal impairment	53 (3.2)
Chronic kidney disease	7 (0.4)
Acute kidney injury	3 (0.2)
Prerenal failure	2 (0.1)
End stage renal disease	1 (0.1)
Hyperkalaemia	29 (1.7)
Blood potassium increased	4 (0.2)
Hyperkalaemia	18 (1.1)
Muscle spasms	7 (0.4)
Dehydration	22 (1.3)
Dehydration	20 (1.2)
Weight decreased	3 (0.2)
Thrombosis and Thromboembolism	8 (0.5)
Cerebellar infarction	1 (0.1)
Cerebral infarction	3 (0.2)
Disseminated intravascular coagulation	1 (0.1)
Pulmonary embolism	2 (0.1)
Retinal artery occlusion	1 (0.1)
Excessive blood pressure reduction	7 (0.4)
Blood pressure decreased	1 (0.1)
Hypotension	4 (0.2)
Orthostatic hypotension	1 (0.1)
Ventricular extrasystoles	1 (0.1)
Gout and Hyperuricemia	151 (9.0)
Blood uric acid increased	12 (0.7)
Gout	3 (0.2)
Hyperuricemia	138 (8.3)
Blood uric acid abnormal	1 (0.1)
Dizziness	25 (1.5)
Dizziness	21 (1.3)
Dizziness postural	4 (0.2)
Diabetes and Hyperglycemia	3 (0.2)
Blood glucose increased	1 (0.1)
Diabetes mellitus	1 (0.1)
Glucose urine present	1 (0.1)
Glaucoma	10 (0.6)
Glaucoma	7 (0.4)
Open-angle glaucoma	1 (0.1)
Normal-tension glaucoma	2 (0.1)
Syncope and loss of consciousness	1 (0.1)
Syncope	1 (0.1)

MedDRA Ver. 25.1

^a^Hepatic disorder

**Table 5:** Patients with increased ALT over 30 IU/L whose levels were below 30 IU/L at baseline

	n (%)
Number of patients	1492
Total patients with increased ALT of > 30 IU/L ^*a*^	571 (38.3)
> 30 and ≤ 90 IU/L	442 (29.6)
> 90 and ≤ 240 IU/L	91 (6.1)
> 240 IU/L	38 (2.5)

^a^Maximum ALT levels during the surveillance

*ALT* Alanine transaminase

### Efficacy

**Fig. 4:**
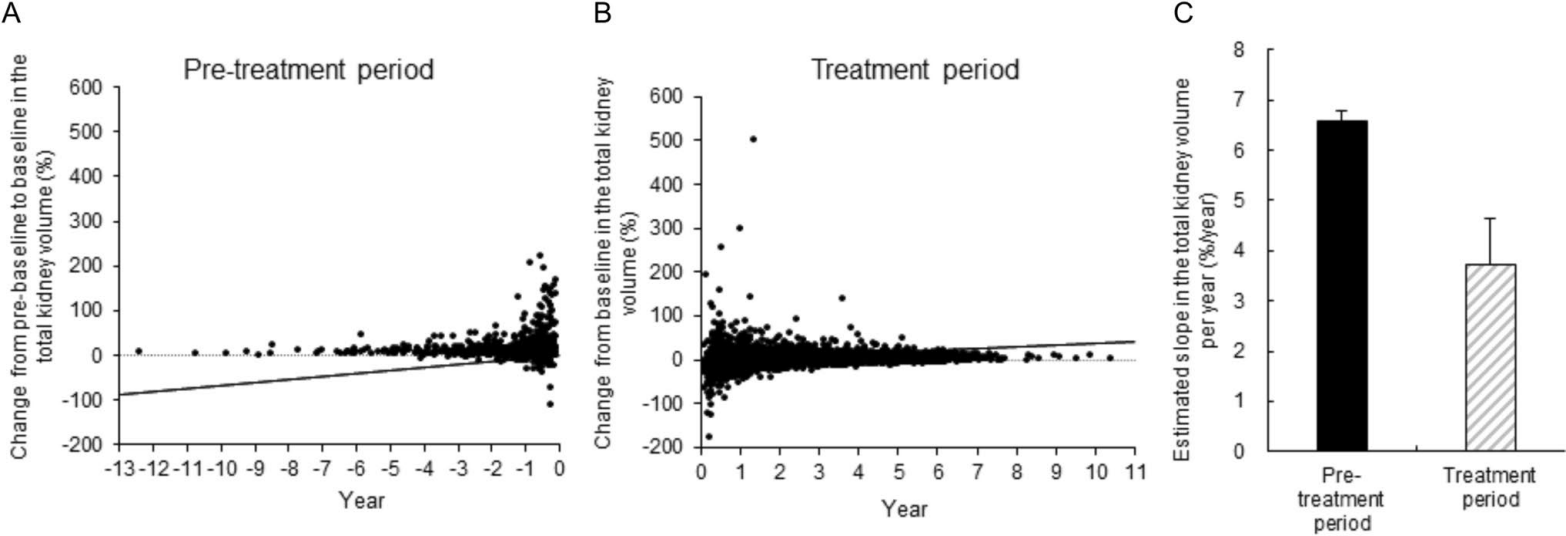
Effect of tolvaptan on TKV. **A** Scatter plot of the estimated slope of TKV during the pre-treatment period, **B** Scatter plot of the estimated slope during the treatment period, **C** Comparison of the estimated percentage change in the TKV slope between pre-treatment period (black) and treatment period (grey) (*P* = 0.0020). The estimated slope was calculated based on a mixed effect model with fixed factors of pre-treatment/treatment group, time, and subject; pre-treatment/treatment period time interaction and subject time interaction as covariates. TKV, total kidney volume

**Fig. 5:**
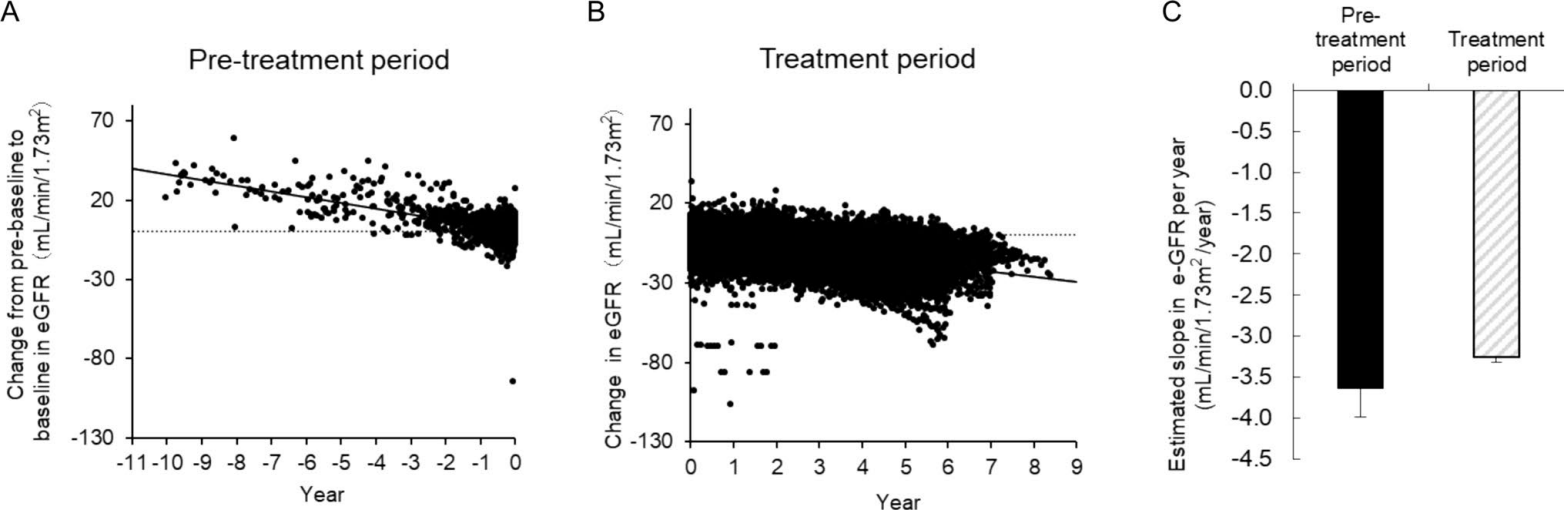
Effect of tolvaptan on eGFR. **A** Scatter plot of the estimated slope of eGFR during the pre-treatment period, **B** Scatter plot of the estimated slope of eGFR during the treatment period, **C** Comparison of the estimated percentage change in the eGFR slope between pre-treatment period (black) and treatment period (grey) (*P* = 0.2728). The estimated slope was calculated based on a mixed effects model with fixed factors of pre-treatment/treatment group, time, and subject; pre-treatment/treatment period time interaction and subject time interaction as covariates. eGFR, estimated glomerular filtration rate

The mean estimated slope of TKV was 6.58%/year (n = 814) before administration with tolvaptan (pre-treatment period) and 3.71%/year (n = 1368) during the administration (treatment period) (Fig. 4). The treatment difference was 0.9730, which was statically significant (Wald test: *P* = 0.0020). The mean estimated slope of the eGFR was −3.63 mL/min/1.73 m^2^/year (n = 1034) in the pre-treatment period and −3.26 mL/min/1.73 m^2^/year (n = 1626) during the treatment period (Fig. 5). The treatment difference was 0.371, which was not statically significant (Wald test: P = 0.2728). The estimated slope of TKV (n = 715) and eGFR (n = 1034) in the pre-treatment and treatment period in patients for which both pre- and post-dose data were available is shown in the Supplementary Figs. S14 and S15, respectively. Estimated slope of the TKV in the pre-treatment and treatment periods with tolvaptan, classified by the CKD stage, is shown in the Supplementary Fig. S16.

## Discussion

During this eight-year surveillance (SLOW-PKD surveillance), we evaluated the safety and efficacy of tolvaptan in real-world settings based on PMS data collected between March 2014 and March 2022. The observed safety and efficacy are comparable to those of two previous, pivotal randomized control trials, TEMPO 3:4 and REPRISE, even though enrolled patients had more advanced disease than that seen in TEMPO 3:4, in terms of TKV and eGFR [10]. Patients had eGFRs similar to those of REPRISE [11]. The mean ± SD eGFR and height-adjusted total kidney volume in this survey were 44.4 ± 21.7 mL/min/1.73 m^2^ and 1241 ± 762 ml/m, respectively, whereas those in the TEMPO 3:4 trial were 81.4 ± 21.0 mL/min/1.73 m^2^ and 979 ± 515 mL/m, respectively [10]. The duration of tolvaptan administration (seven yeas) was the longest compared with TEMPO 3:4 (three years) and REPRISE (one year).

The overall safety profile of tolvaptan was consistent with that seen in previous studies [10, 11]. In other words, AEs in this surveillance and TEMPO study occurred in 65.0% (1086 in 1672 patients) and 97.9% (941 in 961 patients), respectively. The incidence of ADRs was 46.5% (777/1672 patients), and did not exceed 88.6% (851/961 patients) in clinical trials up to the time of approval (data not shown). There was no tendency for the incidence of ADRs and ADRs of special interest to increase after treatment for more than 36 months during the clinical trial (data not shown). This suggests that long-term administration of 3 to 7 years caused no additional safety issues that exceeded levels observed during administration of up to 3 years, and no additional safety management measures would appear to be necessary. Common ADRs were hepatic function abnormal, hyperuricaemia, thirst, hypernatraemia, liver disorder and renal impairment. Among the common ADRs, the incidence of ADRs related to hypernatraemia in clinical trials up to the time of approval was 3.85% (37/961 patients), which was higher in this survey compared to the clinical trials [17]. The presence of liver disease and severity of eGFR were factors affecting liver-related ADRs. It is however unclear whether the severity of eGFR impairment or other factors influenced the risk of liver injury, as patient background factors associated with severity of eGFR were not available. In the REPRISE study, the elevation of ALT to more than three times the ULN was observed in 5.6% of patients in the tolvaptan group [11]. It appears that during our surveillance, the incidence of ALT elevation over 90 IU/L was higher than that of the REPRISE study. In the TEMPO study, most ALT elevations exceeding three times the ULN were observed between 3 and 14 months after the start of administration of tolvaptan, and there were few increments after 15 months. However, in this survey, about 20% of patients with ALT elevation showed an increase after 15 months. This was not planed in this survey, but a Hepatic Adjudication Committee (HAC) consisting of independent hepatic pathologists was established to evaluate hepatic dysfunction observed in this survey, as one of our safety monitoring activities, following the EU Risk Management Plan (RMP), where the relationship between hepatic dysfunction and tolvaptan use was re-evaluated according to the defined method. The committee concluded that the findings in cases with increased ALT after 15 months were not suggestive of a relationship with tolvaptan. We assumed a long-term impact of tolvaptan administration could not be ruled out. After analyzing a database of clinical trials, Watkins et al. [18] reported that liver injury was observed between 3 and 18 months after initiation of tolvaptan treatment. These findings suggest that periodic monitoring of ALT should be considered to allow early detection of acute hepatic failure and hepatic function disorders. Due to limited eligibility criteria, there was a critical lack of information in the clinical trials regarding ADRs in advanced ADPKD (creatinine clearance < 60 mL/min), older ADPKD patients, and those on long-term treatment with tolvaptan. Furthermore, in the package insert of tolvaptan, use is contraindicated in patients with chronic hepatitis, drug-induced liver dysfunction, or a history of hepatic impairment. The package insert recommends that concomitant use of CYP3A4 inhibitors should be avoided. In this surveillance, no increase in the incidence of ADRs was observed in these patients, suggesting the absence of safety concerns, so no new measures were deemed necessary.

Efficacy of tolvaptan for the treatment of ADPKD was confirmed in two randomized clinical trials, TEMPO 3:4 [10] and REPRISE [11]. TEMPO 3:4 was a pivotal study demonstrating the efficacy and safety of tolvaptan over a three-year treatment period. However, the study had certain limitations due to the inclusion criteria, age and disease stage. On the other hand, the REPRISE study focused on the later-stage ADPKD patients, i.e., including stage G4. The REPRISE study demonstrated that tolvaptan was effective in terms of the change in eGFR from baseline. However, change in TKV was not assessed. Real-world data are, therefore, important in understanding the risk/benefit profile of tolvaptan.

In the TEMPO study, the estimated slope of TKV increase in the placebo and tolvaptan groups was 5.51%/year (n = 465) and 2.80%/year (n = 842), respectively, indicating significant suppression of TKV growth (Wald test; P < 0.001). The estimated slope of TKV during this surveillance was 3.71%/year on treatment with tolvaptan, compared with 6.58%/year prior to treatment. It was not possible to directly compare this surveillance with the TEMPO study because of differences in patient baselines and background factors such as TKV; however, the estimated increase in slope of TKV was suppressed during our surveillance. In the TEMPO study, the estimated slope of eGFR in the placebo and tolvaptan groups was −3.70 (n = 484) and − 2.72 (n = 961) mL/min/1.73 m^2^/year, respectively (Wald test; P < 0.001). For our surveillance, the estimated slope of eGFR in the pre-treatment and treatment periods was −3.63 and −3.26 mL/min/1.73 m^2^/year, respectively. Suppression of the eGFR slope decline during our surveillance was weaker than in the TEMPO study, but a certain suppressive effect was observed. In the TEMPO study, the end of the eGFR reduction phase was used as the baseline, in order to exclude the effect of the initial dip, which is a reversible decrease in eGFR immediately after administration. On the other hand, for this surveillance, all eGFR data during the administration period were adopted. Therefore, there is a possibility that the inhibitory effect on the estimated slope of eGFR during the administration period was reduced due to the initial dip of eGFR immediately after administration. Moreover, the dose of tolvaptan should be considered as a factor. The mean dose used in TEMPO 3:4 was 95 mg/day in 88% of patients [10]. In REPRISE, 82.3% of patients were treated with a dose of 120 mg/day [11]. When compared to the randomized clinical trials, much lower doses were used to treat patients during this surveillance.

In conclusion, the safety and efficacy of tolvaptan in patients with ADPKD were assessed in real-world conditions. Comparable safety and efficacy for TKV was observed in relation to the previous two, pivotal randomized control trials. In particular, it seems that the frequency of liver injury was similar to that of the REPRISE study. In order to detect hepatic impairment earlier, we consider it important to continue monitoring hepatic function during treatment with tolvaptan for ADPKD.

## Supplementary Information

Below is the link to the electronic supplementary material.

Supplementary file1 Table S1 Patients characteristics in population of TKV analysis, by CKD stage. Table S2 Serious adverse drug reactions (≥ 2 events). Table S3 Factors influencing onset of liver disorder-related adverse drug reactions (Logistic regression analysis). Fig. S1 Incidence of thirst as an adverse drug reaction of special interest, by onset time. Fig. S2 Incidence of hypernatraemia as an adverse drug reaction of special interest, by onset time. Fig. S3 Incidence of dehydration as an adverse drug reaction of special interest, by onset time. Fig. S4 Incidence of thrombosis and thromboembolism as adverse drug reactions of special interest, by onset time. Fig. S5 Incidence of renal failure and impairment as adverse drug reactions of special interest, by onset time. Fig. S6 Incidence of acute hepatic failure and hepatic function disorder as adverse drug reactions of special interest, by onset time. Fig. S7 Incidence of excessive blood pressure reduction, ventricular fibrillation and ventricular tachycardia as adverse drug reactions of special interest, by onset time. Fig. S8 Incidence of gout and hyperuricaemia as adverse drug reactions of special interest, by onset time. Fig. S9 Incidence of dizziness as an adverse drug reaction of special interest, by onset time. Fig. S10 Incidence of hyperkalaemia as an adverse drug reaction of special interest, by onset time. Fig. S11 Incidence of diabetes and hyperglycaemia as adverse drug reactions of special interest, by onset time. Fig. S12 Incidence of glaucoma as an adverse drug reaction of special interest, by onset time. Fig. S13 Incidence of liver dysfunction-related adverse drug reaction, by CKD stage. Fig. S14 Comparison of the estimated percentage change in the TKV slope between pre-treatment period (white) and treatment period (grey) (P = 0.0011) in patients for which both pre- and post-dose data were available. Fig. S15 Comparison of the estimated percentage change in the eGFR slope between pre-treatment period (white) and treatment period (grey) (P = 0.2728) in patients for which both pre- and post-dose data were available. Fig. S16 Estimated changes in the TKV slope, during the pre-treatment and treatment periods with tolvaptan, classified by CKD stage. (DOCX 229 KB)
