# Supplementary material for: Safety and efficacy of tolvaptan in real‑world Japanese patients with autosomal dominant polycystic kidney disease: final results of SLOW‑PKD surveillance
Source: Clin Exp Nephrol. 2025 Feb 14;29(6):807–17. doi: 10.1007/s10157-025-02634-7 (PMC12125073; doi:10.1007/s10157-025-02634-7)
Supplement: Supplementary file 1 — Supplementary file1 Table S1 Patients characteristics in population of TKV analysis, by CKD stage. Table S2 Serious adverse drug reactions (≥ 2 events). Table S3 Factors influencing onset of liver disorder-related adverse drug reactions (Logistic regression analysis). Fig. S1 Incidence of thirst as an adverse drug reaction of special interest, by onset time. Fig. S2 Incidence of hypernatraemia as an adverse drug reaction of special interest, by onset time. Fig. S3 Incidence of dehydration as an adverse drug reaction of special interest, by onset time. Fig. S4 Incidence of thrombosis and thromboembolism as adverse drug reactions of special interest, by onset time. Fig. S5 Incidence of renal failure and impairment as adverse drug reactions of special interest, by onset time. Fig. S6 Incidence of acute hepatic failure and hepatic function disorder as adverse drug reactions of special interest, by onset time. Fig. S7 Incidence of excessive blood pressure reduction, ventricular fibrillation and ventricular tachycardia as adverse drug reactions of special interest, by onset time. Fig. S8 Incidence of gout and hyperuricaemia as adverse drug reactions of special interest, by onset time. Fig. S9 Incidence of dizziness as an adverse drug reaction of special interest, by onset time. Fig. S10 Incidence of hyperkalaemia as an adverse drug reaction of special interest, by onset time. Fig. S11 Incidence of diabetes and hyperglycaemia as adverse drug reactions of special interest, by onset time. Fig. S12 Incidence of glaucoma as an adverse drug reaction of special interest, by onset time. Fig. S13 Incidence of liver dysfunction-related adverse drug reaction, by CKD stage. Fig. S14 Comparison of the estimated percentage change in the TKV slope between pre-treatment period (white) and treatment period (grey) (P = 0.0011) in patients for which both pre- and post-dose data were available. Fig. S15 Comparison of the estimated percentage change in the eGFR slope between pre-treatment per [file 10157_2025_2634_MOESM1_ESM.docx]

**SUPPLEMENTARY INFORMATION**

**Safety and efficacy of** **tolvaptan in real‑world Japanese patients with autosomal dominant polycystic kidney disease: final results of SLOW‑PKD surveillance**

**Clinical and Experimental Nephrology**

Toshio Mochizuki, Satoru Muto, Kyoko Suzue, Satoshi Komaniwa, Toshiki Tanaka, Yasuhiko Fukuta, Yuko Yamashige

Corresponding author

E-mail address: Komaniwa.Satoshi@otsuka.jp, telephone number: +81-6-6943-7722, fax number: +81-6-6942-3692

**Table S1** Patients characteristics in population of TKV analysis, by CKD stage

**Table S2** Serious adverse drug reactions (≥ 2 events)

**Table S3** Factors influencing onset of liver disorder-related adverse drug reactions (Logistic regression analysis)

**Fig. S1** Incidence of thirst as an adverse drug reaction of special interest, by onset time

**Fig. S2** Incidence of hypernatraemia as an adverse drug reaction of special interest, by onset time

**Fig. S3** Incidence of dehydration as an adverse drug reaction of special interest, by onset time

**Fig. S4** Incidence of thrombosis and thromboembolism as adverse drug reactions of special interest, by onset time

**Fig. S5** Incidence of renal failure and impairment as adverse drug reactions of special interest, by onset time

**Fig. S6** Incidence of acute hepatic failure and hepatic function disorder as adverse drug reactions of special interest, by onset time

**Fig. S7** Incidence of excessive blood pressure reduction, ventricular fibrillation and ventricular tachycardia as adverse drug reactions of special interest, by onset time

**Fig. S8** Incidence of gout and hyperuricaemia as adverse drug reactions of special interest, by onset time

**Fig. S9** Incidence of dizziness as an adverse drug reaction of special interest, by onset time

**Fig. S10** Incidence of hyperkalaemia as an adverse drug reaction of special interest, by onset time

**Fig. S11** Incidence of diabetes and hyperglycaemia as adverse drug reactions of special interest, by onset time

**Fig. S12** Incidence of glaucoma as an adverse drug reaction of special interest, by onset time

**Fig. S13** Incidence of liver dysfunction-related adverse drug reaction, by CKD stage

**Fig. S14** Comparison of the estimated percentage change in the TKV slope between pre-treatment period (white) and treatment period (grey) (P = 0.0011) in patients for which both pre- and post-dose data were available

**Fig. S15** Comparison of the estimated percentage change in the eGFR slope between pre-treatment period (white) and treatment period (grey) (P = 0.2728) in patients for which both pre- and post-dose data were available

**Fig. S16** Estimated changes in the TKV slope, during the pre-treatment and treatment periods with tolvaptan, classified by CKD stage

**Table S1** Patients characteristics in population of TKV analysis, by CKD stage

|  |  | CKD Stage | | | | | |
| --- | --- | --- | --- | --- | --- | --- | --- |
|  |  | Total | G1/G2 | G3a | G3b | G4 | G5 |
| Number of patients | | 1670 | 371 | 336 | 452 | 501 | 10 |
| Sex | |  |  |  |  |  |  |
|  | Male, n (%) | 865 (51.8) | 178 (48.0) | 166 (49.4) | 259 (57.3) | 257 (51.3) | 5 (50.0) |
|  | Female, n (%) | 805 (48.2) | 193 (52.0) | 170 (50.6) | 193 (42.7) | 244 (48.7) | 5 (50.0) |
| Age (years) | |  |  |  |  |  |  |
|  | n | 1670 | 371 | 336 | 452 | 501 | 10 |
|  | Mean ± SD | 49.7 ± 11.2 | 41.9 ± 8.8 | 48.0 ± 9.6 | 52.1 ± 10.6 | 54.3 ± 10.8 | 62.2 ± 11.9 |
| Height (cm) | |  |  |  |  |  |  |
|  | n | 1414 | 312 | 287 | 399 | 409 | 7 |
|  | Mean ± SD | 165.7 ± 9.1 | 166.0 ± 8.7 | 165.9 ± 9.5 | 166.4 ± 9.3 | 164.6 ± 8.8 | 165.7 ± 9.0 |
| Weight (kg) | |  |  |  |  |  |  |
|  | n | 1404 | 308 | 286 | 391 | 413 | 6 |
|  | Mean ± SD | 64.2 ± 12.7 | 63.1 ± 12.0 | 64.6 ± 12.7 | 65.2 ± 12.8 | 64.0 ± 13.1 | 62.3 ± 5.6 |
| BMI (kg/m^2^) | |  |  |  |  |  |  |
|  | n | 1373 | 303 | 279 | 384 | 401 | 6 |
|  | Mean ± SD | 23.3 ± 3.5 | 22.8 ± 3.3 | 23.3 ± 3.5 | 23.4 ± 3.6 | 23.4 ± 3.7 | 23.3 ± 0.9 |
| Systolic blood pressure (mmHg) | |  |  |  |  |  |  |
|  | n | 1427 | 314 | 289 | 398 | 417 | 9 |
|  | Mean ± SD | 130.7 ± 15.8 | 129.6 ± 14.4 | 128.6 ± 15.8 | 131.8 ± 17.0 | 132.1 ± 15.5 | 127.6 ± 15.4 |
| Diastolic blood pressure (mmHg) | |  |  |  |  |  |  |
|  | n | 1426 | 313 | 289 | 398 | 417 | 9 |
|  | Mean ± SD | 82.0 ± 11.9 | 82.1 ± 12.6 | 82.0 ± 11.4 | 81.7 ± 12.4 | 82.2 ± 11.4 | 75.4 ± 11.3 |
| Blood urea nitrogen (mg/dL) | |  |  |  |  |  |  |
|  | n | 1626 | 357 | 326 | 447 | 486 | 10 |
|  | Mean ± SD | 23.9 ± 9.5 | 15.8 ± 4.2 | 19.2 ± 4.2 | 23.4 ± 5.4 | 32.8 ± 9.5 | 51.9 ± 12.2 |
| Serum creatinine (mg/dL) | |  |  |  |  |  |  |
|  | n | 1639 | 365 | 328 | 449 | 487 | 10 |
|  | Mean ± SD | 1.5 ± 0.7 | 0.8 ± 0.2 | 1.1 ± 0.2 | 1.5 ± 0.3 | 2.3 ± 0.6 | 3.7 ± 0.8 |
| eGFR (mL/min/1.73 m^2^) | |  |  |  |  |  |  |
|  | n | 1639 | 365 | 328 | 449 | 487 | 10 |
|  | Mean ± SD | 44.4 ± 21.7 | 76.7 ± 14.4 | 52.1 ± 4.3 | 37.1 ± 4.5 | 22.4 ± 4.4 | 12.7 ± 2.9 |
| Total kidney volume (mL) | |  |  |  |  |  |  |
|  | n | 1637 | 366 | 332 | 444 | 486 | 9 |
|  | Mean ± SD | 2063 ± 1286 | 1458 ± 753 | 1819 ± 980 | 2218 ± 1384 | 2523 ± 1454 | 3218 ± 1926 |
| Height-adjusted total kidney volume (mL/m) | |  |  |  |  |  |  |
|  | n | 1389 | 308 | 284 | 391 | 399 | 7 |
|  | Mean ± SD | 1241 ± 762 | 870 ± 382 | 1081 ± 575 | 1343 ± 840 | 1523 ± 854 | 2252 ± 1269 |
| Total administration period (days) | |  |  |  |  |  |  |
|  | n | 1666 | 368 | 336 | 451 | 501 | 10 |
|  | Mean ± SD | 1320 ± 764 | 1528 ± 750 | 1581 ± 744 | 1427 ± 730 | 915 ± 641 | 502 ± 579 |
| Starting dose (mg) | |  |  |  |  |  |  |
|  | n | 1670 | 371 | 336 | 452 | 501 | 10 |
|  | Mean ± SD | 47.2 ± 17.8 | 51.9 ± 15.1 | 51.1 ± 16.1 | 49.0 ± 16.4 | 39.5 ± 19.2 | 36.0 ± 21.4 |
| Daily dose (mg/day) | |  |  |  |  |  |  |
|  | n | 1665 | 367 | 336 | 451 | 501 | 10 |
|  | Mean ± SD | 66.2 ± 27.9 | 74.6 ± 26.0 | 74.4 ± 26.8 | 69.6 ± 26.2 | 52.1 ± 25.9 | 39.4 ± 18.8 |
| Most frequent daily dose (mg/day) | |  |  |  |  |  |  |
|  | n | 1665 | 367 | 336 | 451 | 501 | 10 |
|  | Mean ± SD | 68.4 ± 32.0 | 77.0 ± 30.3 | 77.4 ± 31.8 | 72.1 ± 30.9 | 53.2 ± 28.8 | 39.0 ± 19.0 |
| Final daily dose (mg/day) | |  |  |  |  |  |  |
|  | n | 1670 | 371 | 336 | 452 | 501 | 10 |
|  | Mean ± SD | 68.0 ± 36.0 | 80.3 ± 32.7 | 80.4 ± 34.0 | 71.8 ± 34.6 | 47.6 ± 31.8 | 40.5 ± 18.8 |

*BMI* body mass index; *CKD* chronic kidney disease; *eGFR* estimated glomerular filtration rate; *SD* standard deviation; *TKV* total kidney volume

**Table S2** Serious adverse drug reactions (≥ 2 events)

|  | Number of patients | | 1672 | |
| --- | --- | --- | --- | --- |
|  | Serious adverse drug reactions, n (%) | | 83 (5.0) | |
|  |  | Hepatic function abnormal | 13 | (0.8) |
|  |  | Liver disorder | 8 | (0.5) |
|  |  | Renal cyst infection | 5 | (0.3) |
|  |  | Renal failure | 5 | (0.3) |
|  |  | Drug-induced liver injury | 3 | (0.2) |
|  |  | Renal cyst haemorrhage | 3 | (0.2) |
|  |  | Renal impairment | 3 | (0.2) |
|  |  | Peritonitis | 2 | (0.1) |
|  |  | Dehydration | 2 | (0.1) |
|  |  | Decreased appetite | 2 | (0.1) |
|  |  | Cerebral infarction | 2 | (0.1) |
|  |  | Subarachnoid haemorrhage | 2 | (0.1) |
|  |  | Chronic kidney disease | 2 | (0.1) |
|  |  | Blood osmolarity increased | 2 | (0.1) |
|  |  | Hepatic enzyme increased | 2 | (0.1) |
|  |  |  |  | MedDRA/J version 25.1 |

**Table S3** Factors influencing onset of liver disorder-related adverse drug reactions (Logistic regression analysis)

|  | Estimated parameter | Standard error | Wald chi-square test | | Estimated odds ratio | | | |
| --- | --- | --- | --- | --- | --- | --- | --- | --- |
|  |  |  | Chi-square value | *P*-value | Odds ratio | 95% Confidence interval | | |
| Intercept | 9.6939 | 6.5218 | 2.2093 | 0.137 | － |  | － |  |
| Sex | 0.0393 | 0.2074 | 0.0358 | 0.850 | 1.040 | 0.693 | － | 1.562 |
| Age | 0.0135 | 0.0150 | 0.8081 | 0.369 | 1.014 | 0.984 | － | 1.044 |
| Age at diagnosis | -0.0015 | 0.0121 | 0.0162 | 0.899 | 0.998 | 0.975 | － | 1.022 |
| Severity (CKD Stage) | -0.2593 | 0.0931 | 7.7511 | 0.005 | 0.772 | 0.643 | － | 0.926 |
| Family history/kidney disease including dialysis | -0.0562 | 0.2808 | 0.0401 | 0.841 | 0.945 | 0.545 | － | 1.639 |
| Family history/intracranial hemorrhage or cerebrovascular event | -0.3938 | 0.2354 | 2.7997 | 0.094 | 0.674 | 0.425 | － | 1.070 |
| Complications/liver disease at the start of administration^a^ | 0.5203 | 0.2010 | 6.7032 | 0.010 | 1.683 | 1.135 | － | 2.495 |
| Complications/hypertension at the start of administration | 0.0055 | 0.3099 | 0.0003 | 0.986 | 1.005 | 0.548 | － | 1.846 |
| Complications/diabetes mellitus at the start of administration | 0.1645 | 0.4554 | 0.1305 | 0.718 | 1.179 | 0.483 | － | 2.878 |
| Complications/hyperlipidemia at the start of administration | -0.0333 | 0.2276 | 0.0214 | 0.884 | 0.967 | 0.619 | － | 1.511 |
| Concomitant medications/CYP3A4 inhibitors | 0.7310 | 0.5497 | 1.7688 | 0.184 | 2.077 | 0.707 | － | 6.101 |
| Body mass index (kg/m^2^) before the start of administration | 0.0231 | 0.0315 | 0.5364 | 0.464 | 1.023 | 0.962 | － | 1.089 |
| Serum Na (mEq/L) before the start of administration | -0.0837 | 0.0463 | 3.2681 | 0.071 | 0.920 | 0.840 | － | 1.007 |

^a^ Complications/liver disease indicates cases where the investigator recorded liver disease on the survey form as a complication at the start of tolvaptan administration

In the case of a yes/no item, the odds ratio of yes to no is indicated. In the case of sex, the odds ratio of male to female is indicated. Odds ratio for a one-unit change in explanatory variable with three or more ordered categories is indicated.

*CKD* chronic kidney disease

Serious adverse drug reaction

Non-serious adverse drug reaction

|  |  | Within  7 d | ≥8 d to  ≤14 d | ≥15 d to  ≤21 d | ≥22 d to  ≤3 m | >3 m to  ≤6 m | >6 m to  ≤9 m | > 9 m to  ≤12 m | > 12 m to  ≤15 m | > 15 m to  ≤36 m | > 36 m to  ≤48 m | > 48 m |
| --- | --- | --- | --- | --- | --- | --- | --- | --- | --- | --- | --- | --- |
| n (%) | Serious | 0 (0.0) | 0 (0.0) | 0 (0.0) | 0 (0.0) | 0 (0.0) | 0 (0.0) | 0 (0.0) | 0 (0.0) | 0 (0.0) | 0 (0.0) | 0 (0.0) |
|  | Non-serious | 91 (66.9) | 5 (3.7) | 10 (7.4) | 10 (7.4) | 12 (8.8) | 4 (2.9) | 1 (0.7) | 0 (0.0) | 1 (0.7) | 0 (0.0) | 2 (1.5) |

**Fig. S1** Incidence of thirst as an adverse drug reaction of special interest, by onset time

Serious adverse drug reaction

Non-serious adverse drug reaction

Serious adverse drug reaction

Non-serious adverse drug reaction

|  |  | Within 7 d | ≥8 d to | ≥15 d to | ≥ 22 d to | > 3 m to | > 6 m to | > 9 m to | > 12 m to | > 15 m to | > 36 m to | > 48 m |
| --- | --- | --- | --- | --- | --- | --- | --- | --- | --- | --- | --- | --- |
|  |  |  | ≤14 d | ≤21 d | ≤3 m | ≤6 m | ≤9 m | ≤12 m | ≤15 m | ≤36 m | ≤48 m |  |
| n (%) | Serious | 0 (0.0) | 0 (0.0) | 0 (0.0) | 0 (0.0) | 0 (0.0) | 0 (0.0) | 0 (0.0) | 0 (0.0) | 0 (0.0) | 0 (0.0) | 0 (0.0) |
|  | Non-serious | 35 (25.9) | 8 (5.9) | 3 (2.2) | 12 (8.9) | 14 (10.4) | 5 (3.7) | 5 (3.7) | 2 (1.5) | 25 (18.5) | 13 (9.6) | 13 (9.6) |

**Fig. S2** Incidence of hypernatraemia as an adverse drug reaction of special interest, by onset time

Serious adverse drug reaction

Non-serious adverse drug reaction

|  |  | Within 7 d | ≥8 d to | ≥15 d to | ≥ 22 d to | > 3 m to | > 6 m to | > 9 m to | > 12 m to | > 15 m to | > 36 m to | > 48 m |
| --- | --- | --- | --- | --- | --- | --- | --- | --- | --- | --- | --- | --- |
|  |  |  | ≤14 d | ≤21 d | ≤3 m | ≤6 m | ≤9 m | ≤12 m | ≤15 m | ≤36 m | ≤48 m |  |
| n (%) | Serious | 1 (4.0) | 0 (0.0) | 0 (0.0) | 0 (0.0) | 0 (0.0) | 0 (0.0) | 0 (0.0) | 0 (0.0) | 1 (4.0) | 0 (0.0) | 0 (0.0) |
|  | Non-serious | 3 (12.0) | 1 (4.0) | 0 | 3 (12.0) | 2 (8.0) | 0 | 2 (8.0) | 1 (4.0) | 7 (28.0) | 3 (12.0) | 1 (4.0) |

**Fig. S3** Incidence of dehydration as an adverse drug reaction of special interest, by onset time

Serious adverse drug reaction

Non-serious adverse drug reaction

|  |  | Within 7 d | ≥8 d to | ≥15 d to | ≥ 22 d to | > 3 m to | > 6 m to | > 9 m to | > 12 m to | > 15 m to | > 36 m to | > 48 m |
| --- | --- | --- | --- | --- | --- | --- | --- | --- | --- | --- | --- | --- |
|  |  |  | ≤14 d | ≤21 d | ≤3 m | ≤6 m | ≤9 m | ≤12 m | ≤15 m | ≤36 m | ≤48 m |  |
| n (%) | Serious | 0 (0.0) | 0 (0.0) | 0 (0.0) | 0 (0.0) | 3 (37.5) | 2 (25.0) | 0 (0.0) | 0 (0.0) | 0 (0.0) | 0 (0.0) | 0 (0.0) |
|  | Non-serious | 0 (0.0) | 0 (0.0) | 0 (0.0) | 0 (0.0) | 0 (0.0) | 1 (12.5) | 0 (0.0) | 0 (0.0) | 1 (12.5) | 1 (12.5) | 0 (0.0) |

**Fig. S4** Incidence of thrombosis and thromboembolism as adverse drug reactions of special interest, by onset time

Serious adverse drug reaction

Non-serious adverse drug reaction

|  |  | Within 7 d | ≥8 d to | ≥15 d to | ≥ 22 d to | > 3 m to | > 6 m to | > 9 m to | > 12 m to | > 15 m to | > 36 m to | > 48 m |
| --- | --- | --- | --- | --- | --- | --- | --- | --- | --- | --- | --- | --- |
|  |  |  | ≤14 d | ≤21 d | ≤3 m | ≤6 m | ≤9 m | ≤12 m | ≤15 m | ≤36 m | ≤48 m |  |
| n (%) | Serious | 0 (0.0) | 0 (0.0) | 0 (0.0) | 1 (0.8) | 2 (1.6) | 1 (0.8) | 1 (0.8) | 0 (0.0) | 6 (4.8) | 1 (0.8) | 0 (0.0) |
|  | Non-serious | 8 (6.4) | 3 (2.4) | 4 (3.2) | 9 (7.2) | 6 (4.8) | 13 (10.4) | 9 (7.2) | 3 (2.4) | 38 (30.4) | 8 (6.4) | 12 (9.6) |

**Fig. S5** Incidence of renal failure and impairment as adverse drug reactions of special interest, by onset time

Serious adverse drug reaction

Non-serious adverse drug reaction

|  |  | Within 7 d | ≥8 d to | ≥15 d to | ≥ 22 d to | > 3 m to | > 6 m to | > 9 m to | > 12 m to | > 15 m to | > 36 m to | > 48 m |
| --- | --- | --- | --- | --- | --- | --- | --- | --- | --- | --- | --- | --- |
|  |  |  | ≤14 d | ≤21 d | ≤3 m | ≤6 m | ≤9 m | ≤12 m | ≤15 m | ≤36 m | ≤48 m |  |
| n (%) | Serious | 1 (0.3) | 0 (0.0) | 0 (0.0) | 9 (2.7) | 9 (2.7) | 4 (1.2) | 4 (1.2) | 0 (0.0) | 3 (0.9) | 1 (0.3) | 0 (0.0) |
|  | Non-serious | 11 (3.3) | 3 (0.9) | 5 (1.5) | 48 (14.4) | 77 (23.1) | 48 (14.4) | 21 (6.3) | 14 (4.2) | 48 (14.4) | 14 (4.2) | 13 (3.9) |

**Fig. S6** Incidence of acute hepatic failure and hepatic function disorder as adverse drug reactions of special interest, by onset time

Serious adverse drug reaction

Non-serious adverse drug reaction

|  |  | Within 7 d | ≥8 d to | ≥15 d to | ≥ 22 d to | > 3 m to | > 6 m to | > 9 m to | > 12 m to | > 15 m to | > 36 m to | > 48 m |
| --- | --- | --- | --- | --- | --- | --- | --- | --- | --- | --- | --- | --- |
|  |  |  | ≤14 d | ≤21 d | ≤3 m | ≤6 m | ≤9 m | ≤12 m | ≤15 m | ≤36 m | ≤48 m |  |
| n (%) | Serious | 0 (0.0) | 0 (0.0) | 0 (0.0) | 0 (0.0) | 0 (0.0) | 0 (0.0) | 0 (0.0) | 0 (0.0) | 1 (16.7) | 0 (0.0) | 0 (0.0) |
|  | Non-serious | 0 (0.0) | 0 (0.0) | 0 (0.0) | 2 (33.3) | 0 (0.0) | 2 (33.3) | 0 (0.0) | 0 (0.0) | 1 (16.7) | 0 (0.0) | 0 (0.0) |

**Fig. S7** Incidence of excessive blood pressure reduction, ventricular fibrillation and ventricular tachycardia as adverse drug reactions of special interest, by onset time

Serious adverse drug reaction

Non-serious adverse drug reaction

|  |  | Within 7 d | ≥8 d to | ≥15 d to | ≥ 22 d to | > 3 m to | > 6 m to | > 9 m to | > 12 m to | > 15 m to | > 36 m to | > 48 m |
| --- | --- | --- | --- | --- | --- | --- | --- | --- | --- | --- | --- | --- |
|  |  |  | ≤14 d | ≤21 d | ≤3 m | ≤6 m | ≤9 m | ≤12 m | ≤15 m | ≤36 m | ≤48 m |  |
| n (%) | Serious | 0 (0.0) | 0 (0.0) | 0 (0.0) | 0 (0.0) | 0 (0.0) | 0 (0.0) | 0 (0.0) | 0 (0.0) | 0 (0.0) | 0 (0.0) | 0 (0.0) |
|  | Non-serious | 29 (17.9) | 11 (6.8) | 9 (5.6) | 33 (20.4) | 13 (8.0) | 15 (9.3) | 12 (7.4) | 5 (3.1) | 22 (13.6) | 5 (3.1) | 8 (4.9) |

**Fig. S8** Incidence of gout and hyperuricaemia as adverse drug reactions of special interest, by onset time

Serious adverse drug reaction

Non-serious adverse drug reaction

|  |  | Within 7 d | ≥8 d to | ≥15 d to | ≥ 22 d to | > 3 m to | > 6 m to | > 9 m to | > 12 m to | > 15 m to | > 36 m to | > 48 m |
| --- | --- | --- | --- | --- | --- | --- | --- | --- | --- | --- | --- | --- |
|  |  |  | ≤14 d | ≤21 d | ≤3 m | ≤6 m | ≤9 m | ≤12 m | ≤15 m | ≤36 m | ≤48 m |  |
| n (%) | Serious | 0 (0.0) | 0 (0.0) | 0 (0.0) | 0 (0.0) | 0 (0.0) | 0 (0.0) | 0 (0.0) | 0 (0.0) | 0 (0.0) | 0 (0.0) | 0 (0.0) |
|  | Non-serious | 2 (8.0) | 1 (4.0) | 1 (4.0) | 2 (8.0) | 3 (12.0) | 5 (20.0) | 3 (12.0) | 2 (8.0) | 5 (20.0) | 1 (4.0) | 0 (0.0) |

**Fig. S9** Incidence of dizziness as an adverse drug reaction of special interest, by onset time

Serious adverse drug reaction

Non-serious adverse drug reaction

|  |  | Within 7 d | ≥8 d to | ≥15 d to | ≥ 22 d to | > 3 m to | > 6 m to | > 9 m to | > 12 m to | > 15 m to | > 36 m to | > 48 m |
| --- | --- | --- | --- | --- | --- | --- | --- | --- | --- | --- | --- | --- |
|  |  |  | ≤14 d | ≤21 d | ≤3 m | ≤6 m | ≤9 m | ≤12 m | ≤15 m | ≤36 m | ≤48 m |  |
| n (%) | Serious | 0 (0.0) | 0 (0.0) | 0 (0.0) | 0 (0.0) | 0 (0.0) | 0 (0.0) | 0 (0.0) | 0 (0.0) | 0 (0.0) | 0 (0.0) | 0 (0.0) |
|  | Non-serious | 2 (5.9) | 1 (2.9) | 0 (0.0) | 2 (5.9) | 6 (17.7) | 3 (8.8) | 2 (5.9) | 0 (0.0) | 11 (32.4) | 3 (8.8) | 4 (11.8) |

**Fig. S10** Incidence of hyperkalaemia as an adverse drug reaction of special interest, by onset time

Serious adverse drug reaction

Non-serious adverse drug reaction

|  |  | Within 7 d | ≥8 d to | ≥15 d to | ≥ 22 d to | > 3 m to | > 6 m to | > 9 m to | > 12 m to | > 15 m to | > 36 m to | > 48 m |
| --- | --- | --- | --- | --- | --- | --- | --- | --- | --- | --- | --- | --- |
|  |  |  | ≤14 d | ≤21 d | ≤3 m | ≤6 m | ≤9 m | ≤12 m | ≤15 m | ≤36 m | ≤48 m |  |
| n (%) | Serious | 0 (0.0) | 0 (0.0) | 0 (0.0) | 0 (0.0) | 0 (0.0) | 0 (0.0) | 0 (0.0) | 0 (0.0) | 0 (0.0) | 0 (0.0) | 0 (0.0) |
|  | Non-serious | 0 (0.0) | 0 (0.0) | 0 (0.0) | 0 (0.0) | 0 (0.0) | 1 (33.3) | 0 (0.0) | 0 (0.0) | 2 (66.7) | 0 (0.0) | 0 (0.0) |

**Fig. S11** Incidence of diabetes and hyperglycaemia as adverse drug reactions of special interest, by onset time

Serious adverse drug reaction

Non-serious adverse drug reaction

|  |  | Within 7 d | ≥8 d to | ≥15 d to | ≥ 22 d to | > 3 m to | > 6 m to | > 9 m to | > 12 m to | > 15 m to | > 36 m to | > 48 m |
| --- | --- | --- | --- | --- | --- | --- | --- | --- | --- | --- | --- | --- |
|  |  |  | ≤14 d | ≤21 d | ≤3 m | ≤6 m | ≤9 m | ≤12 m | ≤15 m | ≤36 m | ≤48 m |  |
| n (%) | Serious | 0 (0.0) | 0 (0.0) | 0 (0.0) | 0 (0.0) | 0 (0.0) | 0 (0.0) | 0 (0.0) | 0 (0.0) | 0 (0.0) | 0 (0.0) | 0 (0.0) |
|  | Non-serious | 1 (12.5) | 0 (0.0) | 0 (0.0) | 1 (12.5) | 1 (12.5) | 1 (12.5) | 1 (12.5) | 0 (0.0) | 1 (12.5) | 0 (0.0) | 2 (25.0) |

**Fig. S12** Incidence of glaucoma as an adverse drug reaction of special interest, by onset time

|  |  |  | | liver-related ADRs | |
| --- | --- | --- | --- | --- | --- |
|  |  | N | n | | % |
| CKD Stage | G1 | 63 | 12 | | 19.05 |
|  | G2 | 308 | 60 | | 19.48 |
|  | G3a | 336 | 52 | | 15.48 |
|  | G3b | 452 | 71 | | 15.71 |
|  | G4 | 501 | 67 | | 13.37 |
|  | G5 | 10 | 0 | | 0 |

**Fig. S13** Incidence of liver dysfunction-related adverse drug reaction, by CKD stage

*ADR* adverse drug reactions, *CKD* chronic kidney disease

Estimated slope in the total kidney volume per year (%/year)

**Fig. S14** Comparison of the estimated percentage change in the TKV slope between pre-treatment period (white) and treatment period (grey) (P = 0.0011) in patients for which both pre- and post-dose data were available

Estimated slope in e-GFR per year (mL/min/1.73 m^2^ /year)

**Fig. S15** Comparison of the estimated percentage change in the eGFR slope between pre-treatment period (white) and treatment period (grey) (P = 0.2728) in patients for which both pre- and post-dose data were available

Change in the estimated slope of the total kidney volume (%)

|  |  | No. of patients | |  |
| --- | --- | --- | --- | --- |
|  |  | Pre-treatment  period | Treatment  period | *P* value |
| CKD Stage | G1/G2 | 200 | 319 | 0.0040 |
|  | G3a | 172 | 288 | 0.0189 |
|  | G3b | 218 | 374 | 0.9359 |
|  | G4 | 221 | 380 | 0.2818 |
|  | G5 | 3 | 5 | <0.0001 |
|  | Total | 814 | 1368 | 0.0020 |

**Fig. S16** Estimated changes in the TKV slope, during the pre-treatment and treatment periods with tolvaptan, classified by CKD stage

*CKD* chronic kidney disease
